# Hsa_circ_0018909 promotes non-small cell lung cancer by directly regulating hsa-miR-513b-5p

**DOI:** 10.3389/fonc.2025.1542742

**Published:** 2025-06-23

**Authors:** Qi Liu, Yong-Kui Yu, Hao-Miao Li, Wei Wang, Jing Xu

**Affiliations:** ^1^ Department of Cardiovascular Surgery, The First Affiliated Hospital of Zhengzhou University, Zhengzhou, China; ^2^ Department of Thoracic Surgery, Zhengzhou University Affiliated Cancer Hospital, Zhengzhou, China; ^3^ Department of Thoracic Surgery, Henan Cancer Hospital, Zhengzhou, China

**Keywords:** non-small cell lung cancer, hsa_circ_0018909, hsa-miR-513b-5p, proliferation, migration, invasion

## Abstract

**Background:**

Circular RNAs (circRNAs) regulate gene expression by functioning as competing endogenous RNAs (ceRNAs) and are increasingly recognized for their involvement in cancer progression. Hsa_circ_0018909 is upregulated in non-small cell lung cancer (NSCLC); however, its functional role and underlying mechanisms remain poorly understood. This study aimed to investigate the role of hsa_circ_0018909 and its downstream regulatory axis in NSCLC.

**Methods:**

Differentially expressed circRNAs were identified from the GSE101586 dataset. The expression levels of hsa_circ_0018909 and hsa-miR-513b-5p were validated in NSCLC tissues and cell lines. CircBase and UCSC were used to determine the genomic origin of hsa_circ_0018909, and its subcellular localization was examined using fluorescence *in situ* hybridization. The interaction between hsa_circ_0018909 and hsa-miR-513b-5p was predicted with TargetScan and verified through dual-luciferase reporter assays. Functional assays, including CCK-8, colony formation, Transwell migration/invasion, and subcutaneous tumor formation in nude mice, were conducted to evaluate the effects of hsa_circ_0018909. Rescue experiments and Western blot analyses were performed to identify downstream targets and elucidate the underlying regulatory mechanisms.

**Results:**

Among the five upregulated circRNAs identified, only hsa_circ_0018909 consistently showed high expression in NSCLC tissues and cell lines and was associated with shorter overall and disease-free survival. Hsa_circ_0018909 was primarily localized in the cytoplasm. Overexpression of hsa_circ_0018909 enhanced the proliferation, migration, and invasion of NSCLC cells, while its knockdown produced the opposite effects. Hsa-miR-513b-5p mimics attenuated the oncogenic effects of hsa_circ_0018909, whereas inhibition of hsa-miR-513b-5p reversed the suppressive effects of hsa_circ_0018909 knockdown. Mechanistically, hsa_circ_0018909 directly binds to hsa-miR-513b-5p and negatively regulates its expression. Malate dehydrogenase 1 (MDH1) was identified as a direct downstream target of hsa-miR-513b-5p. Rescue experiments confirmed that MDH1 mediates the tumor-promoting effects resulting from hsa-miR-513b-5p inhibition in NSCLC cells.

**Conclusion:**

Hsa_circ_0018909 is significantly upregulated in NSCLC and promotes tumor progression by sponging hsa-miR-513b-5p, thereby indirectly upregulating its downstream target, MDH1. These findings suggest that the hsa_circ_0018909/miR-513b-5p/MDH1 axis may represent a potential therapeutic target for NSCLC.

## 1 Introduction

Lung cancer is a global health concern and remains the leading cause of cancer-related deaths (1). Over the past 10 years, the number of new lung cancer cases in the USA has declined by 1%–3% annually (2). According to the American Cancer Society, there were still an estimated 236,740 new lung cancer cases in 2022 (2). Regrettably, about half of the 2 million new lung cancer cases worldwide occur in Asia (3). In addition, the lung cancer mortality rate in the USA has steadily decreased from 1990 to 2022, with an estimated 130,180 deaths reported in 2022. However, the number of new lung cancer cases continues to rise in China (4). Among these cases, non-small cell lung cancer (NSCLC) accounts for 80%–85% (5). Currently, treatment options for NSCLC include surgery, radiotherapy, chemotherapy, immunotherapy, and molecular-targeted therapy (6). Among them, molecularly targeted therapies—serving as a model of precision medicine—have been developed for NSCLC subtypes defined by specific oncogenic driver mutations, leading to improved outcomes for patients with NSCLC (7). Therefore, further research on biomarkers is essential to enhance the effectiveness of targeted therapies in treating NSCLC.

Circular RNAs (circRNAs) are a type of noncoding RNA discovered over 40 years ago. They are produced through a covalent linkage formed by back-splicing of linear RNA (8). The covalently closed loop structure of circRNAs led to their widespread underestimation in earlier transcriptome analyses (9). Fortunately, with the development of specific biochemical and computational bioinformatics methods, numerous circRNAs have since been identified across various cell lines and species (9). While most circRNAs are expressed at low levels, some are more abundant than their linear counterparts (10). Among highly expressed circRNAs, most are processed from internal exons of premessenger RNA and include multiple exons (11). Due to their high abundance, stability, long half-life, resistance to degradation, tissue- and developmental stage-specific expression patterns, and widespread presence in various bodily fluids, circRNAs have great potential as diagnostic biomarkers or therapeutic targets (12). Notably, when circRNA markers are combined with traditional tumor markers, the sensitivity and specificity of diagnosis and treatment efficacy analysis are significantly improved (13).

CircRNAs participate in gene regulation through various mechanisms, including sponging microRNAs (miRNAs) to regulate mRNA transcription and protein translation, as well as interacting with RNA-binding proteins (14). The adsorbed miRNAs lead to the degradation or translational inhibition of their target transcripts. This process, known as the competing endogenous RNA (ceRNA) mechanism, may have the potential a diagnostic marker or therapeutic target in various diseases (15). Many circRNAs have been implicated in cancer through the ceRNA mechanism, including lung, liver, and bladder cancers (16–18). For instance, circRNA DNA polymerase alpha 2, accessory subunit (POLA2) and G protein subunit beta 1 mRNA are highly expressed in lung cancer tissues and are associated with poor prognosis. Moreover, circRNA POLA2 regulates G protein subunit beta 1 expression by sponging miR-326, thereby contributing to the stemness and proliferation of cancer cells (16). CircRNA 104348 is significantly upregulated in liver cancer and is associated with poor prognosis. This circRNA regulates the proliferation, migration, invasion, and apoptosis of liver cancer cells by directly targeting miR-187-3p and may influence the Wnt/β-catenin pathway and rhotekin 2 expression (18). Similarly, Circ_0058063 is overexpressed in bladder cancer, where it promotes cell proliferation and invasion while inhibiting apoptosis by competitively binding forkhead box P4 to miR-486-3p (17).

Based on the analysis of Gene Expression Omnibus (GEO) data, five upregulated circRNAs were identified from GSE101586 using the following criteria: (1) the top 20 upregulated circRNAs, (2) circRNAs with a length between 500 and 1,000 bp, and (3) no studies on their mechanisms in NSCLC published by 2022. The expression levels of these five circRNAs were then validated in human NSCLC tissues (*N* = 10) and adjacent normal tissues (*N* = 10). Among them, only hsa_circ_0018909 was significantly upregulated in NSCLC tissues compared to adjacent normal tissues. Moreover, previous studies have reported that hsa_circ_0018909 is expressed at low levels in colorectal cancer but is highly expressed in NSCLC (19, 20). Only one study has shown that circ_0018909 is highly expressed in pancreatic cancer cells and that its knockdown significantly inhibits cell growth and metastasis while increasing apoptosis by regulating miR-545-3p (21). Therefore, our study focused on the potential ceRNA mechanism of hsa_circ_0018909 in NSCLC. This study verified the function of hsa_circ_0018909 through both *in vitro* and *in vivo* experiments and further explored its potential ceRNA mechanism. Furthermore, we analyzed the relationship between the expression of hsa_circ_0018909 and its target miRNAs in relation to clinical prognosis. To our knowledge, this is the first study to investigate the mechanism of hsa_circ_0018909 in NSCLC, providing a new perspective on potential therapeutic targets and prognostic indicators for NSCLC.

## 2 Materials and methods

### 2.1 Selection of circRNAs

In this study, five upregulated circRNAs were identified from the GSE101586 dataset (five pairs of tissues) in GEO (https://www.ncbi.nlm.nih.gov/geo/) based on the following criteria: first, circRNAs ranked among the top 20 upregulated circRNAs; second, circRNAs with lengths ranging from 500 to 1,000 bp were specifically selected, as circRNAs of this moderate length typically exhibit greater structural stability and efficient circularization. Previous studies have also indicated that circRNAs within this size range commonly function via ceRNA regulatory mechanisms (8, 12); third, circRNAs whose mechanisms in NSCLC had already been reported before 2022 were excluded. These differentially expressed circRNAs (*p* < 0.05 and |Log2 fold change| > 1) were identified in NSCLC (20).

### 2.2 NSCLC tissue collection

**Figure 1:**
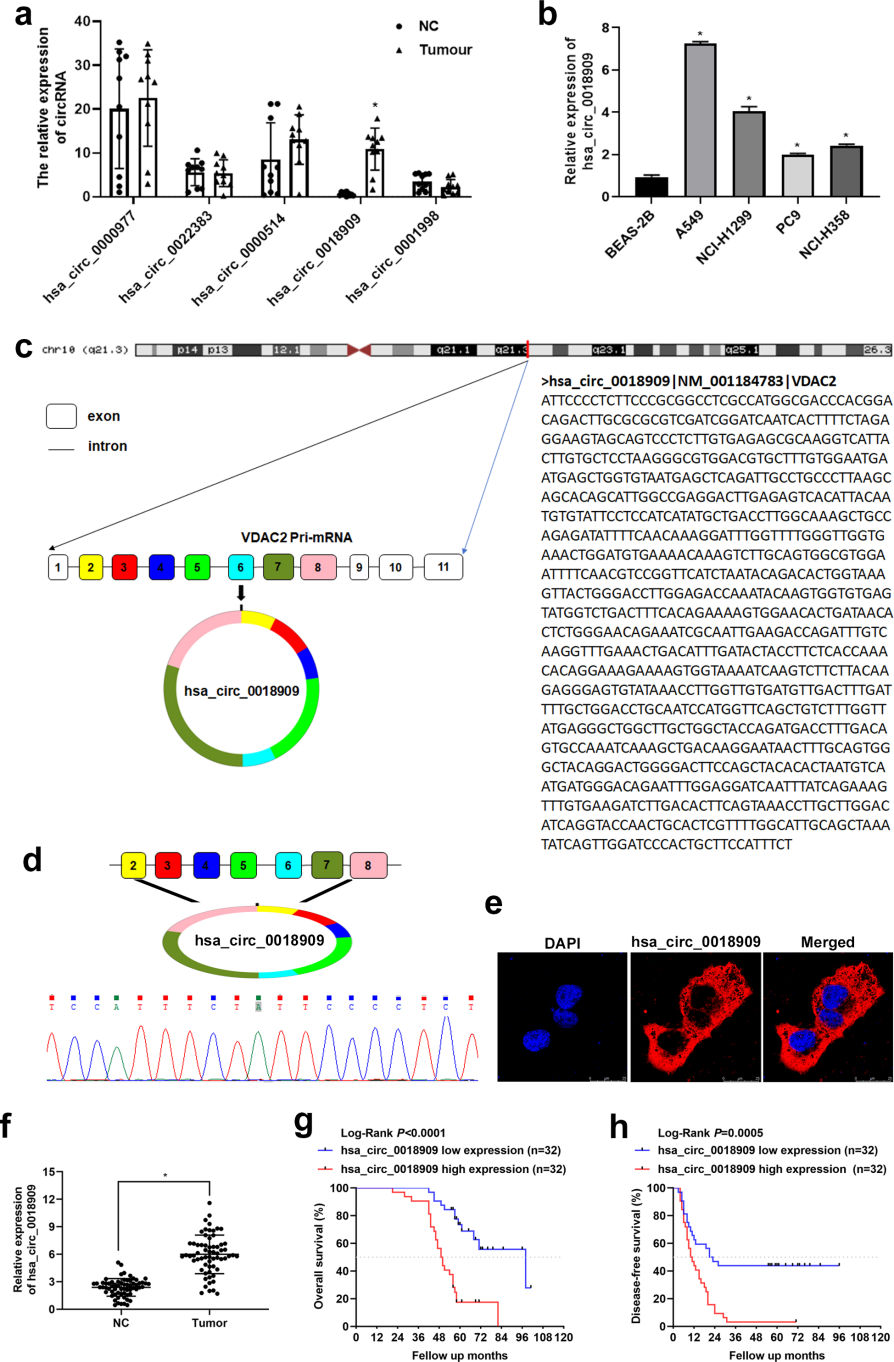
Validation of circRNA expression in non-small cell lung cancer (NSCLC) tissues and cells. **(a)** The expression of hsa_circ_0000977, hsa_circ_0022383, hsa_circ_0004104, hsa_circ_0018909, and hsa_circ_0001998 was determined in NSCLC (tumor) and adjacent normal tissue (NC) samples by quantitative reverse transcriptase polymerase chain reaction. *N* = 20. **(b)** The expression of hsa_circ_0018909 was measured in normal human lung epithelial cells (BEAS-2B) and four NSCLC cell lines, including A549, NCI-H1299, PC9, and NCI-H358, using quantitative reverse transcriptase polymerase chain reaction. *N* = 3. **(c)** CircBase combined with University of California-Santa Cruz analysis was used to determine the origin of hsa_circ_0018909. **(d)** Total RNA from multiple NSCLC and adjacent normal tissues was extracted, and amplified products obtained from the mixed total RNA samples were analyzed to determine the splice junction of hsa_circ_0018909 using Sanger sequencing. **(e)** Fluorescence *in situ* hybridization detection of the subcellular localization of hsa_circ_0018909 in A549 cells. **(f)** The expression of hsa_circ_0018909 in NC and tumor tissues was measured using a quantitative reverse transcriptase polymerase chain reaction. *N* = 128. **(g)** Based on the expression of hsa_circ_001890, Kaplan–Meier overall survival curves for patients with NSCLC were plotted. **(h)** Based on the expression of hsa_circ_001890, Kaplan–Meier disease-free survival curves for patients with NSCLC were plotted. VDAC2, voltage-dependent anion channel 2; DAPI, 4′,6-diamidino-2-phenylindole. ^*^ *p* < 0.05.

Human NSCLC tissues (*N* = 10) and adjacent normal tissues (*N* = 10) were collected to validate the expression of five upregulated circRNAs shown in **Figure 1**. An additional set of human NSCLC tissues (*N* = 64) and adjacent normal tissues (*N* = 64) was collected to assess the expression levels of hsa_circ_0018909 and hsa-miR-513b-5p. The 10 tissue pairs used for validation in **Figure 1** were not included in the 64 pairs used for further analysis. This study was conducted in accordance with the principles of the Declaration of Helsinki and was reviewed and approved by the ethics committee of the First Affiliated Hospital of Zhengzhou University (Approval Number: 2024-KY-0078-001, dated 23 August 2024).

### 2.3 Quantitative reverse transcriptase polymerase chain reaction

In our study, quantitative reverse transcriptase polymerase chain reaction (qRT-PCR) was used to detect the expression of circRNAs and miRNAs in tissues and cells. U6 was the internal control of miRNAs, and GAPDH served as the internal reference for circRNAs. The primer sequences are listed in **Table 1**. Briefly, the experimental procedure includes the following steps: first, total RNA was extracted from NSCLC tissues (100 mg) or cells (5 × 10^6^) according to the instructions provided with TriQuick Reagent (Solarbio, No. R1100, Beijing, China). Subsequently, total RNA (1 μg) was reverse transcribed into complementary DNA (cDNA) using the HiScript III RT SuperMix for qPCR (+gDNA wiper, No. R323-01; Vazyme, Nanjing, China), according to the manufacturer’s instructions. Finally, expression levels were measured using the ChamQ Universal SYBR qPCR Master Mix (Vazyme, No. Q711-02) with 1 μL of cDNA, following the standard protocol. Relative expression levels were calculated using the 2^−△△CT^ method.

**Table 1:** Primer sequences tested in this study.

Primer name	Sequence (5′-3′)
miR-125b-2-3p-RT	GTCGTATCCAGTGCAGGGTCCGAGGTATTCGCACTGGATACGACgtccca
miR-125b-2-3p-F	TCACAAGTCAGGCTCTTGGGAC
miR-643-RT	GTCGTATCCAGTGCAGGGTCCGAGGTATTCGCACTGGATACGACctacct
miR-643-F	ACTTGTATGCTAGCTCAGGTAG
miR-1255b-2-3p-RT	GTCGTATCCAGTGCAGGGTCCGAGGTATTCGCACTGGATACGACtggatg
miR-1255b-2-3p-F	AACCACTTTCTTTGCTCATCCA
miR-513b-5p-RT	GTCGTATCCAGTGCAGGGTCCGAGGTATTCGCACTGGATACGACataaat
miR-513b-5p-F	TTCACAAGGAGGTGTCATTTAT
miR-3200-3p-RT	GTCGTATCCAGTGCAGGGTCCGAGGTATTCGCACTGGATACGACcagacc
miR-3200-3p-F	CACCTTGCGCTACTCAGGTCTG
Tniverse-R	GTGCAGGGTCCGAGGT
T6-F	CTCGCTTCGGCAGCACA
T6-R	AACGCTTCACGAATTTGCGT
hsa_circ_0018909-F	AACCTTGCTTGGACATCAGG
hsa_circ_0018909-R	TTCCTCTAGAAAAGTGATTGATCC
GAPDH-F	GAGTCAACGGATTTGGTCGT
GAPDH-R	GACAAGCTTCCCGTTCTCAG

### 2.4 Splice junction analysis of hsa_circ_0018909 and validation

The qPCR products of hsa_circ_0018909 were sent to Sangon Biotech (Shanghai, China) for Sanger sequencing. The sequence data were visualized using Chromas 2.6.6 (Technelysium Pty Ltd., Australia, Brisbane). Based on CircBase and the University of California Santa Cruz (UCSC) genome database, the splice junction of hsa_circ_0018909 was confirmed through analysis of the Sanger sequencing results.

### 2.5 Cell culture

Human normal lung epithelial cells (BEAS-2B, No. CC4011), NSCLC cell lines (A549 [No. CC0202], NCI-H1299 [No. CC0203], PC9 [No. CC0204], NCI-H358 [No. CC0205]), and 293T cells (No. CC4003) were purchased from CellCook (Guangzhou, China). BEAS-2B cells were cultured in Airway-EpiMedium (CellCook, No. CC4011S). A549 cells were cultured in an F-12K medium (CellCook, No. CM2004) supplemented with 10% fetal bovine serum. NCI-H1299, PC9, and NCI-H358 cells were cultured in RPMI-1640 (Gibco, No. 11875, Waltham, Massachusetts, USA) supplemented with 10% fetal bovine serum. 293T cells were cultured in high-glucose DMEM (Gibco, No. 1995) supplemented with 10% fetal bovine serum. All the above cells were maintained in incubators at 37°C with 5% CO_2_ and appropriate humidity.

### 2.6 Fluorescence *in situ* hybridization

Fluorescence *in situ* hybridization (FISH) was performed using a FISH kit (Bersin, Guangzhou, China; No. Bes1001) to determine the subcellular localization of hsa_circ_0018909 in A549 cells. The CY5-labeled probe sequences for hsa_circ_0018909 (AGAGGGGAATAGAAATGGAA-CY5) were synthesized by Generay (Shanghai, China). In brief, A549 cells were seeded onto cell slides and fixed with 4% paraformaldehyde for 10 min. The cells were then incubated with a prehybridization solution at 37°C for 30 min, followed by a hybridization solution at 73°C for 5 min, and finally transferred to 42°C for 20 h. After washing with saline sodium citrate buffer, the A549 cells were incubated with 4′,6-diamidino-2-phenylindole (DAPI) for 10 min. Finally, following two washes with phosphate-buffered saline (PBS) for 5 min each, the signals were observed under a microscope.

### 2.7 Construction of stable strain overexpression or knockdown of hsa_circ_0018909

For the construction of stable cell lines with overexpression or knockdown of hsa_circ_0018909, lentiviruses for hsa_circ_0018909 interference, overexpression, and their respective controls were prepared by Hanheng Biotechnology (Shanghai, China). These lentiviruses were then individually used to infect A549 or PC9 cells to generate the following stable cell lines: A549-shCIRC (a stable strain with silenced expression of hsa_circ_0018909), A549-shNC (negative control stable strain with silenced expression of hsa_circ_0018909), PC9-LVCIRC (stable strain with overexpression of hsa_circ_0018909), and PC9-LVNC (negative control stable strain with overexpression of hsa_circ_0018909), following the protocol provided by Hanheng Biotechnology.

### 2.8 Cell proliferation

In our study, the Cell Counting Kit-8 (CCK-8) and clone formation assay were used to evaluate changes in cell proliferation. For CCK-8 detection, 3,000 cells per well were seeded in 96-well plates. After incubation for the indicated time, CCK-8 regent (Solarbio, No. CA1210) was added to each well for 2 h, and the optical density (OD) at 450 nm was measured using a microplate reader (BioTek ELx808, Winooski, VT, USA). For the clone formation assay, 100 cells/well were seeded in six-well plates with 2 mL of medium/well. After 7 days of cultivation (with the culture medium changed every 2 days), the culture medium was removed from each well. The cells were rinsed twice with PBS and fixed in 4% paraformaldehyde for 15 min, followed by washing twice with PBS. The fixed cells were then stained with 0.1% crystal violet solution (Solarbio) for 20 min and rinsed with running water.

### 2.9 Transwell assay

Transwell assays were used to assess changes in cell migration and invasion. The experimental method was similar to a previous study (22), with a few revisions. For the invasion assay, filters were first precoated with Matrigel (BD, San Jose, CA, USA). The migration and invasion assays were then performed as follows: cells (1 × 10^5^, in 200 μL of serum-free medium) were seeded in the upper chamber, and 500 μL of medium containing 10% fetal bovine serum was added to the lower chamber. After incubation for 18–24  h at 37°C in a humidified incubator with 5% CO_2_, cells remaining on the upper surface of the membrane were removed using a cotton swab. The migrated/invasive cells were fixed with 4% paraformaldehyde for 15 min, then washed twice with PBS. The cells were subsequently stained with 0.1% crystal violet solution for 20 min and washed thrice with PBS. Stained cells were observed under a Nikon Eclipse Ti Inverted Research Microscope (Tokyo, Japan).

### 2.10 Cell transfection

To overexpress or inhibit hsa-miR-513b-5p, hsa-miR-513b-5p mimics, hsa-miR-513b-5p inhibitor, and their respective negative controls (mimics NC and inhibitor NC) were synthesized by Genecefe Biotechnology Co. Ltd (Wuhan, China). These fragments were transfected into cells using Lipofectamine 3000 reagent (Thermo Fisher, Shanghai, China), following the manufacturer’s instructions. To investigate whether malate dehydrogenase 1 (MDH1) is involved in the biological effects of hsa-miR-513b-5p, rescue experiments were performed by co-transfecting A549 cells with si-MDH1 and the hsa-miR-513b-5p inhibitor. The si-MDH1 and negative control siRNA were synthesized by Genecefe Biotechnology (Wuhan, China). Transfections were conducted using Lipofectamine 3000. After 48 h, cells were collected for qRT-PCR, Western blot, proliferation, colony formation, migration, and invasion assays, as described above.

### 2.11 Luciferase activity detection

TargetScan was used to analyze the miRNAs potentially recruited by hsa_circ_0018909 and to predict the binding sites between hsa_circ_0018909 and hsa-miR-513b-5p. To confirm these predicted binding sites, the wild-type (WT) sequences and corresponding mutant (Mut) sequences of hsa_circ_0018909 were synthesized to construct six recombinant plasmids at Genecefe Biotechnology Co. Ltd., including pmirGLO-WT site 1, pmirGLO-WT site 2, pmirGLO-WT site 3, pmirGLO-Mut site 1, pmirGLO-Mut site 2, and pmirGLO-Mut site 3. TargetScan was also applied to predict the binding sites between hsa-miR-513b-5p and the 3′ untranslated region (3′UTR) of MDH1 mRNA. The wild-type (WT) and mutant (Mut) sequences of the MDH1 3′UTR were cloned into pmirGLO reporter vectors, generating pmirGLO-WT-MDH1 and pmirGLO-Mut-MDH1 constructs. All luciferase reporter plasmids were co-transfected with hsa-miR-513b-5p mimics or mimic negative controls (mimics NC) into 293T cells using Lipofectamine 3000 reagent, according to the manufacturer’s instructions. After 48 h of incubation, cells were lysed in 1 × cell lysis buffer, and the supernatant was collected by centrifugation (12,000×*g*, 10 min at 4°C).

For luciferase activity detection, 100 μL of Luciferase Reaction Substrate I was added to the detection tube, followed by the addition of 20 μL of supernatant to measure the activity of the firefly luciferase reporter. Subsequently, 100 μL of Luciferase Reaction Substrate II was added to the same detection tube to detect the activity of the Renilla luciferase reporter. Luciferase activity was calculated as the ratio of firefly luciferase activity to Renilla luciferase activity using the TransDetect^®^ Double-Luciferase Reporter Assay Kit (TransGen, No. FR201-01, Beijing, China).

### 2.12 Subcutaneous tumor formation in nude mice (*in vivo*)

A total of 24 BALB/c nude mice were randomly divided into four groups (six mice per group) after being housed in a specific pathogen-free barrier environment for 1 week. The four groups were as follows: A549-shCIRC, A549-shNC, PC9-LVCIRC, and PC9-LVNC. Each mouse was subcutaneously injected with 3 × 10^6^ cells. Tumor size was recorded each week, and the tumor volume was calculated using the formula: *V* = 1/2 × (length) × (width) × (width). After 4 weeks, all tumor tissues were excised, weighed, and photographed. All animal protocols were approved by the ethical committee of the Guangzhou Forevergen Medical Laboratory Animal Center (Approval Number: IACUC-AEWC-F231106116, dated 6 November 2023). All animal experiments were conducted in accordance with ARRIVE guidelines and the IACUC Handbook (third edition).

### 2.13 Statistical analysis

To analyze the relationship between hsa_circ_0018909 expression and clinicopathological parameters, the Chi-square test or Fisher’s exact test was used. Relative hsa_circ_0018909 expression levels were normalized to those of the corresponding adjacent normal tissues (NC). NSCLC patients were then classified into a high-expression group (relative expression > 2.5-fold) and a low-expression group (< 2.5-fold). Univariate and multivariate analyses were conducted using Cox proportional hazards regression models to identify predictors of overall survival (OS) and disease-free survival (DFS) in NSCLC patients. Variables that showed statistical significance (*p* < 0.05) in the univariate analysis were subsequently included in the multivariate Cox regression model. For categorical variables with three levels, comparisons were made by combining two categories and comparing them against the third. Specifically, pathology grade was compared as high vs. (median + low), lymphatic metastasis as N0 vs. (N1 + N2), and clinical stage as stage I vs. (stage II + III) when calculating hazard ratios (HRs) in both univariate and multivariate analyses. The correlation between the expression levels of hsa_circ_0018909 and hsa-miR-513b-5p was evaluated using Spearman’s correlation analysis. Statistical analyses, unless otherwise specified, were performed using GraphPad Prism 7 software (La Jolla, CA, USA). Student’s *t*-test was used to compare differences between two groups, while one-way analysis of variance (ANOVA) was applied for comparisons among three or more groups. Data are presented as mean ± standard deviation. A two-tailed *p*-value < 0.05 was considered statistically significant.

## 3 Results

### 3.1 Validation of the circRNA expression in NSCLC tissues and cells

As mentioned in the **Introduction** and **Materials and method** sections, five circRNAs—hsa_circ_0000977, hsa_circ_0022383, hsa_circ_0004104, hsa_circ_0018909, and hsa_circ_0001998—were selected from the GSE101586 dataset. To validate their expression in human NSCLC tissues, qRT-PCR was conducted on 10 pairs of clinical samples. The results revealed that only hsa_circ_0018909 was significantly upregulated in NSCLC tissues compared to adjacent normal tissues (**Figure 1a**). Therefore, we further examined hsa_circ_0018909 expression in four NSCLC cell lines (A549, NCI-H1299, PC9, and NCI-H358) and the human normal lung epithelial cell line BEAS-2B. The results showed that hsa_circ_0018909 was significantly upregulated in all NSCLC cell lines compared to BEAS-2B, with expression levels increased by more than twofold (**Figure 1b**). Specifically, A549 showed a sevenfold increase, NCI-H1299 more than a fourfold increase, PC9 more than a twofold increase, and NCI-H358 about a 2.5-fold increase (**Figure 1b**). These findings indicate that hsa_circ_0018909 is highly expressed in NSCLC tissues and cells, consistent with the GSE101586 data. Therefore, we speculated that hsa_circ_0018909 may be involved in the progression of NSCLC.

### 3.2 Hsa_circ_0018909 came from voltage-dependent anion channel 2 mRNA and was mainly localized in the cytoplasm

**Figure 2:**
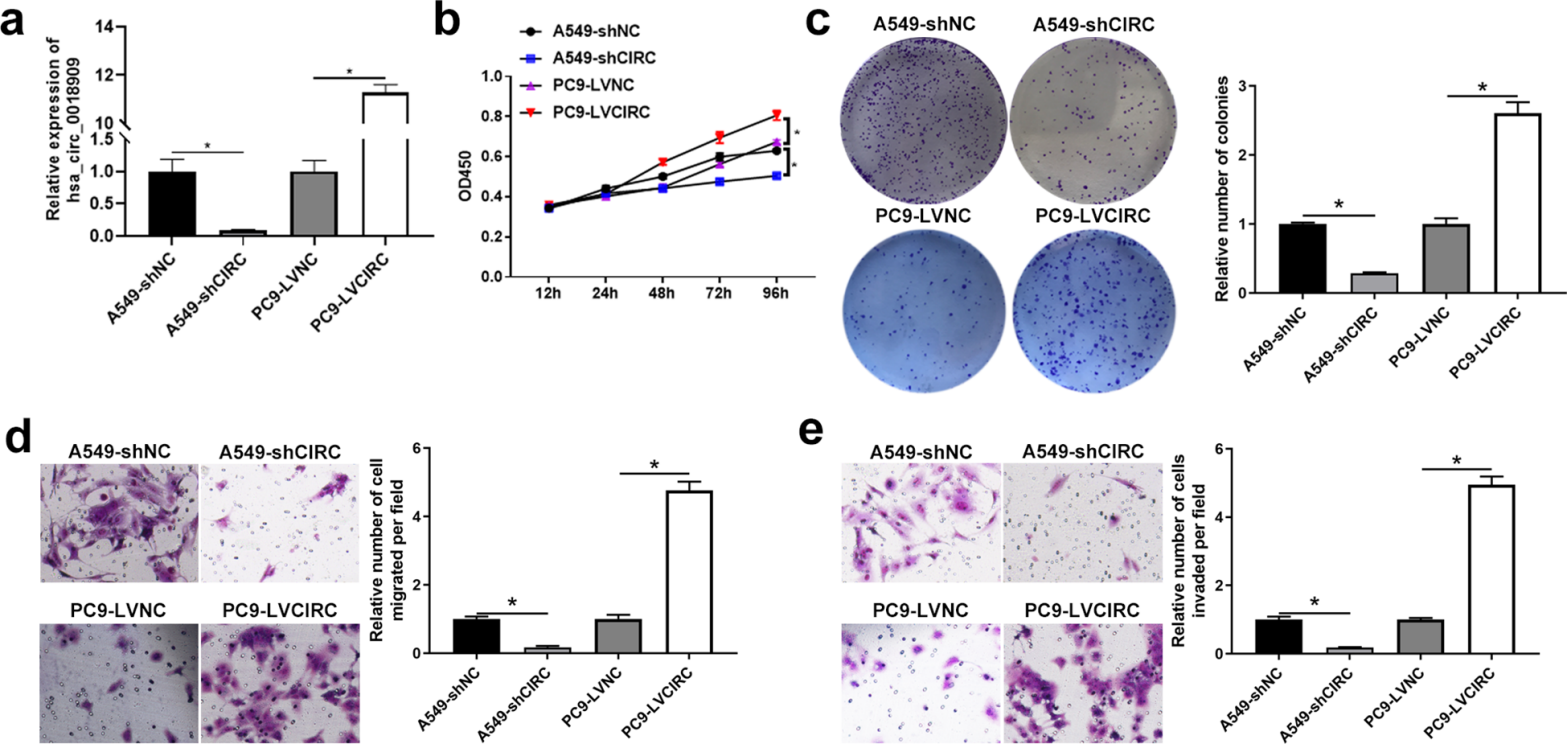
High expression of hsa_circ_0018909 enhanced the cellular function of A549, and low expression of hsa_circ_0018909 reduced the cellular function of PC9 cells. **(a)** Detection of hsa_circ_0018909 expression in A549-shNC, A549-shCIRC, PC9-LVNC, and PC9-LVCIRC using quantitative reverse transcriptase polymerase chain reaction. *N* = 3. **(b)** The proliferation ability of A549-shNC, A549-shCIRC, PC9-LVNC, and PC9-LVCIRC cells was determined using the Cell Counting Kit-8 assay at 12, 24, 48, 72, and 96 h *N* = 3. **(c)** Clone formation assay was used to assess cell proliferation in A549-shNC, A549-shCIRC, PC9-LVNC, and PC9-LVCIRC cells. *N* = 3. **(d, e)** Transwell assay was used to evaluate migration in A549-shNC, A549-shCIRC, PC9-LVNC, and PC9-LVCIRC cells. A549-shCIRC, stable strain with silenced expression of hsa_circ_0018909; A549-shNC, negative control stable strain with silenced expression of hsa_circ_0018909; PC9-LVCIRC, stable strain with overexpression of hsa_circ_0018909; PC9-LVNC, negative control stable strain with overexpression of hsa_circ_0018909. ^*^ *p* < 0.05.

To better investigate the function of hsa_circ_0018909, we first confirmed its circular structure. Analysis using CircBase and UCSC revealed that hsa_circ_0018909 is derived from exons 2/3/4/5/6/7/8 of the mother gene voltage-dependent anion channel 2 (VDAC2) mRNA located on 10 chromosomes (**Figure 1c**). Sanger sequencing of qPCR products from mixed NSCLC and adjacent normal tissues confirmed the presence of a splice junction in hsa_circ_0018909 (**Figures 2c, d**). These results confirmed that hsa_circ_0018909 truly exists in NSCLC. FISH results showed that hsa_circ_0018909 is predominantly localized in the cytoplasm (**Figure 1e**).

### 3.3 High expression of hsa_circ_0018909 in NSCLC was associated with low OS and DFS

Hsa_circ_0018909 expression was then measured in a larger cohort of 128 clinical NSCLC samples. Compared to adjacent normal tissues, hsa_circ_0018909 expression was significantly elevated in NSCLC tumor tissues (**Figure 1f**). Patients with high hsa_circ_0018909 expression showed significantly reduced OS and DFS compared to those with low expression levels (**Figures 1g, h**). Moreover, high hsa_circ_0018909 expression levels were significantly associated with smoking, LN metastasis, and clinical stage (FIGO), but showed no association with age, sex, and pathology grade (**Table 2**). Univariate and multivariate analyses indicated that smoking, hsa_circ_0018909 expression levels, and clinical stage (I vs. II vs. III) were significantly correlated with OS (**Table 3**).

**Table 2:** Relationship between hsa_0018909 expression and clinicopathological parameters.

Variable	Total	Low expression	High expression	*P*-value
Age (year)				
≤ 45	15	10	5	0.140
> 45	49	22	27	
Sex				
Female	35	19	16	0.451
Male	29	13	16	
Smoking				
No	34	24	10	0.000^*^
Yes	30	8	22	
Pathology grade				
Low	21	8	13	0.183
Median or high	43	24	19	
LN metastasis^a^				
N0	26	20	6	0.000^*^
N1 or N2	38	12	26	
Clinical stage (FIGO)				
I	21	17	4	0.001^*^
II or III	43	15	28	

^*^ *p*-value < 0.05: Chi-square (and Fisher’s exact) test.

a American Joint Committee on Cancer (AJCC), patients were staged in accordance with the Eighth Edition of the AJCC Cancer’s TNM Classification.

**Table 3:** Univariate and multivariate analyses of overall survival (OS) in patients with non-small cell lung cancer (*n* = 64).

Variables	Univariate analysis			Multivariate analysis		
	HR	95% CI	*P*-value	HR	95% CI	*P*-value
Age (> 45 vs. ≤ 45)	2.517	1.042–6.082	0.040^*^	2.366	0.887–6.310	0.085
Sex (male vs. female)	0.862	0.450–1.653	0.655	NA	NA	NA
Smoking (yes vs. no)	2.188	1.152–4.154	0.017^*^	0.765	0.340–1.718	0.516^*^
hsa_circ_0018909 expression (high vs. low)	4.668	2.278–9.565	0.000^*^	3.225	1.352–7.693	0.008^*^
Pathology grade (high or median vs. low)	0.710	0.367–1.372	0.308	NA	NA	NA
Lymphatic metastasis (N1 or N2 vs. N0)	8.489	3.429–21.025	0.000^*^	5.926	1.521–23.096	0.010^*^
Clinical stage (II or III vs. I)	6.966	2.608–18.606	0.000^*^	1.114	0.256–4.854	0.886

HR, hazard ratio; *95%* CI, 95% confidence interval. Cox regression analysis. ^*^ *p* < 0.05.

### 3.4 Hsa_circ_0018909 involved in the development of NSCLC cells

Subsequently, the cell function of hsa_circ_0018909 was examined in A549 cells, which exhibit the highest expression of hsa_circ_0018909 among NSCLC cell lines, and in PC9 cells, which show the lowest expression. Short hairpin RNA (shRNA) targeting hsa_circ_0018909 was introduced into A549 cells, and qRT-PCR results confirmed that hsa_circ_0018909 expression was significantly reduced in A549-shCIRC cells compared to A549-shNC cells (**Figure 2a**). Conversely, hsa_circ_0018909 was overexpressed in PC9 cells via plasmid transfection, with qRT-PCR revealing a 10-fold increase in hsa_circ_0018909 expression in PC9-LVCIRC cells compared to PC9-LVNC cells (**Figure 2a**). These results confirmed that hsa_circ_0018909 was successfully knocked down in A549 cells and overexpressed in PC9 cells. Subsequently, cell functions, including proliferation, migration, and invasion, were assessed following modulation of hsa_circ_0018909 levels. Compared to A549-shNC cells, A549-shCIRC cells showed a significant decrease in proliferation at 96 h (**Figure 2b**). Consistent with this, the number of cell clones in A549-shCIRC was reduced to less than half that of A549-shNC (**Figure 2c**). Furthermore, Transwell assays revealed that the migration capacity of A549-shCIRC cells was approximately one-eighth that of A549-shNC cells (**Figure 2d**). In the invasion assay, we also observed that the number of invading cells in A549-shCIRC was reduced to approximately one-eighth of that in A549-shNC cells (**Figure 2e**). In contrast to these findings, PC9-LVNC cells showed a significant increase in proliferation at 96 h compared to PC9-LVCIRC cells, with more than a twofold increase in colony formation and over a fourfold increase in both migration and invasion cell numbers (**Figures 2b–e**).

### 3.5 Hsa_circ_0018909 directly regulated the hsa-miR-513b-5p expression

**Figure 3:**
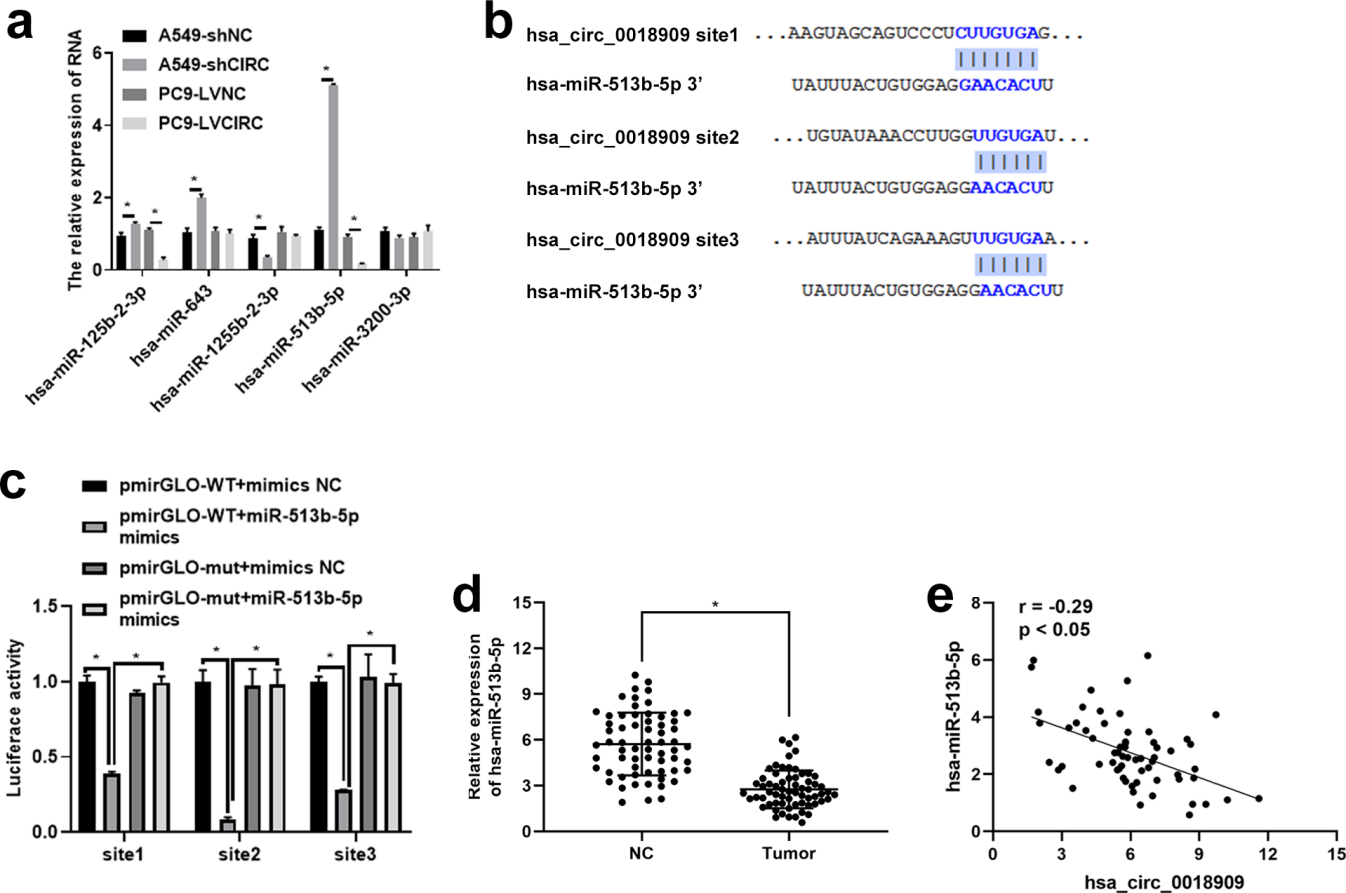
Hsa-circ_0018909 directly targets and binds hsa-miR-513b-5p. **(a)** Detection of the levels of hsa-miR-125b-2-3p, hsa-miR-643, hsa-miR-1255b-2-3p, hsa-miR-513b-5p, and hsa-miR-3200-3p in A549-shNC, A549-shCIRC, PC9 LVNC, and PC9 LVCIRC cells using quantitative reverse transcriptase polymerase chain reaction. **(b)** TargetScan analysis of the binding site between hsa_circ_0018909 and hsa-miR-513b-5p. **(c)** Luciferase activity assay confirmed the binding sites between hsa_circ_0018909 and hsa-miR-513b-5p. **(d)** Detection of hsa-miR-513b-5p levels in NSCLC (tumor) and adjacent normal (NC) tissues. **(e)** The expression correlation between hsa_circ_0018909 and hsa-miR-513b-5p. ^*^ *p* < 0.05.

To further investigate the potential mechanism of hsa_circ_0018909 in NSCLC development, TargetScan was used to analyze the miRNAs that could be recruited by hsa_0018909, with the results shown in **Table 4**. We selected miRNAs with more than two binding sites, including hsa-miR-125b-2-3p, hsa-miR-643, hsa-miR-1255b-2-3p, hsa-miR-513b-5p, and hsa-miR-3200-3p, for further expression analysis in A549-shNC, A549-shCIRC, PC9-LVNC, and PC9-LVCIRC. Compared with A549-shNC, the expression levels of hsa-miR-125b-2-3p, hsa-miR-643, and hsa-miR-513b-5p were significantly increased, while hsa-miR-1255b-2-3p was significantly decreased in A549-shCIRC (**Figure 3a**). In PC9-LVCIRC, hsa-miR-125b-2-3p and hsa-miR-513b-5p were significantly downregulated compared to PC9-LVNC cells (**Figure 3a**). Notably, the expression trends of hsa-miR-125b-2-3p and hsa-miR-513b-5p were opposite between the overexpression hsa_circ_0018909 group and silence of hsa_circ_0018909 group. As the expression changes were more pronounced following either overexpression of hsa_circ_0018909 or knockdown of hsa_circ_0018909, hsa-miR-513b-5p was selected for targeted validation using the luciferase reporter assay. Based on the predicted binding sites shown in **Figure 3b**, we constructed pmirGLO-WT and pmirGLO-mut plasmids for each binding site of hsa_circ_0018909. Luciferase activity was significantly reduced in cells transfected with the wild-type constructs compared to those with pmirGLO-wt + mimics NC or pmirGLO-wt + miR-513b-5p mimics (**Figure 3c**). Luciferase activity showed no significant difference among the three groups: pmirGLO-wt + mimics NC, pmirGLO-mut + mimics NC, and pmirGLO-wt + miR-513b-5p mimics (**Figure 3c**). These results indicate that hsa-circ_0018909 can directly bind to hsa-miR-513b-5p. More importantly, qRT-PCR analysis revealed that hsa-miR-513b-5p expression was significantly lower in NSCLC tissues compared to adjacent normal tissues (**Figure 3d**). Additionally, correlation analysis demonstrated that hsa-circ_0018909 expression was negatively associated with an hsa-miR-513b-5p showed that the expression of hsa_circ_0018909 was negatively associated with hsa-miR-513b-5p expression (**Figure 3e**). The results observed in NSCLC tissues were consistent with those in the NSCLC cells.

**Table 4:** Predicted miRNAs interacting with hsa_circ_0018909 based on TargetScan and CircAtlas databases.

circAltas ID	MicroRNA	TargetScan
hsa_circ_0018909	hsa-miR-145-5p	1
hsa_circ_0018909	hsa-miR-125b-2-3p	2
hsa_circ_0018909	hsa-miR-186-3p	1
hsa_circ_0018909	hsa-miR-194-3p	1
hsa_circ_0018909	hsa-miR-369-5p	1
hsa_circ_0018909	hsa-miR-330-3p	1
hsa_circ_0018909	hsa-miR-452-3p	1
hsa_circ_0018909	hsa-miR-490-5p	1
hsa_circ_0018909	hsa-miR-643	2
hsa_circ_0018909	hsa-miR-1226-3p	1
hsa_circ_0018909	hsa-miR-1288-3p	1
hsa_circ_0018909	hsa-miR-1255b-2-3p	2
hsa_circ_0018909	hsa-miR-513b-5p	3
hsa_circ_0018909	hsa-miR-1910-5p	1
hsa_circ_0018909	hsa-miR-3137	1
hsa_circ_0018909	hsa-miR-3200-3p	2
hsa_circ_0018909	hsa-miR-4303	1
hsa_circ_0018909	hsa-miR-3670	1
hsa_circ_0018909	hsa-miR-4454	1
hsa_circ_0018909	hsa-miR-4736	1

### 3.6 Hsa_circ_0018909 control the proliferation, migration, and invasion ability of NSCLC cells by directly regulating the expression of hsa-miR-513b-5p

**Figure 4:**
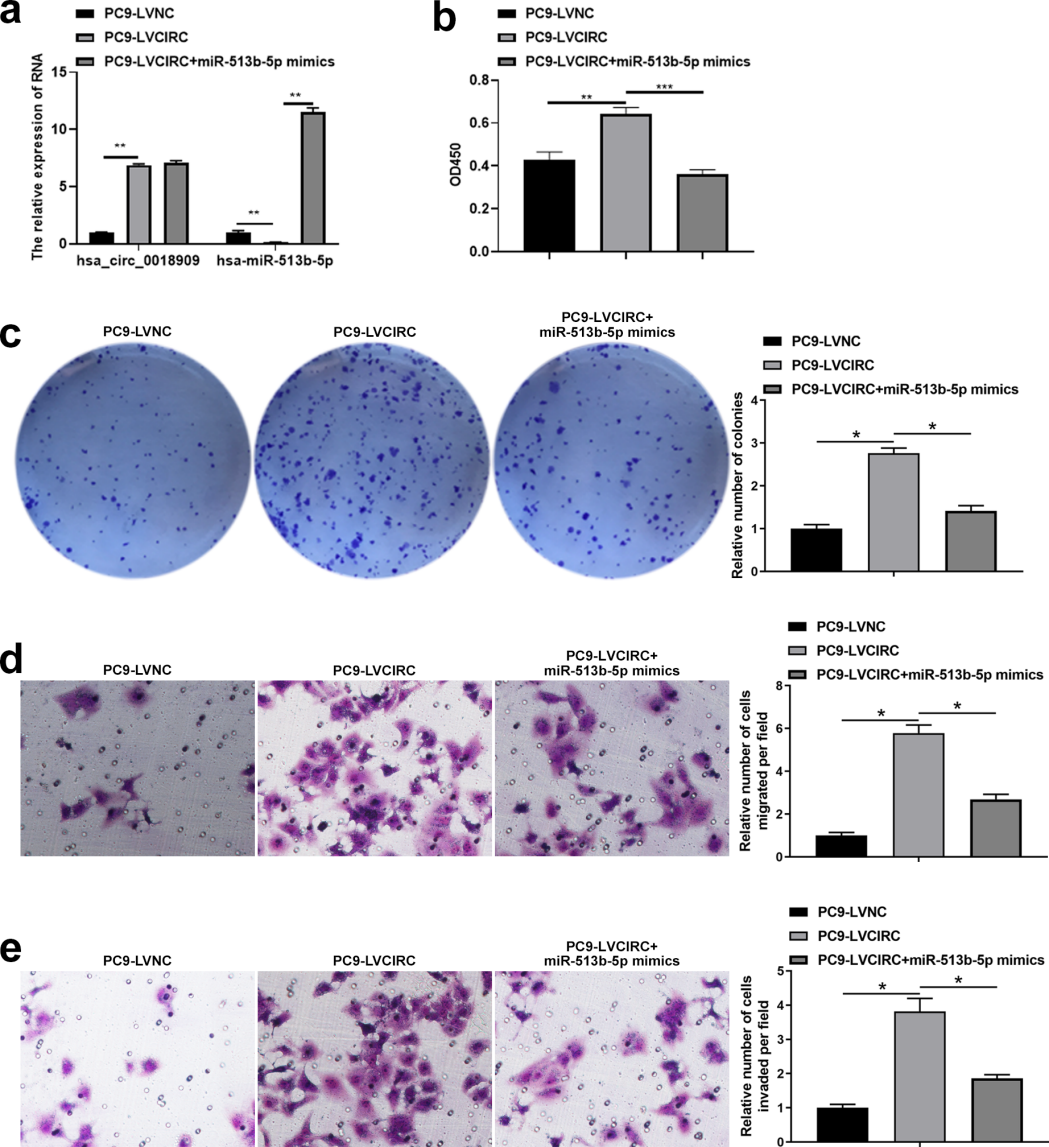
Overexpression of hsa-miR-513b-5p weakened the effect of high levels of hsa_circ_0018909 on the proliferation, migration, and invasion abilities of PC9 cells. Mimic negative control and hsa-miR-513b-5p mimics were transfected into PC9-LVNC and PC9-LVCIRC cells for 48 h, respectively. Experimental groups were divided into PC9-LVNC (PC9-LVNC cells transfected with mimic negative control), PC9-LVCIRC (PC9-LVCIRC cells transfected with mimic negative control), and PC9-LVCIRC + miR-513b-5p mimics (PC9-LVCIRC cells transfected with miR-513b-5p mimics). **(a)** Expression levels of hsa_circ_0018909 and hsa-miR-513b-5p were measured using quantitative reverse transcriptase polymerase chain reaction. **(b)** Cell proliferation was detected using the Cell Counting Kit-8 assay. **(c)** Cell proliferation was assessed using a clone formation assay. Cell migration **(d)** and invasion **(e)** were detected using a Transwell assay. PC9-LVCIRC, stable strain with overexpression of hsa_circ_0018909; PC9-LVNC, negative control stable strain with overexpression of hsa_circ_0018909. ^*^ *p* < 0.05, **p < 0.01, ***p < 0.001.

**Figure 5:**
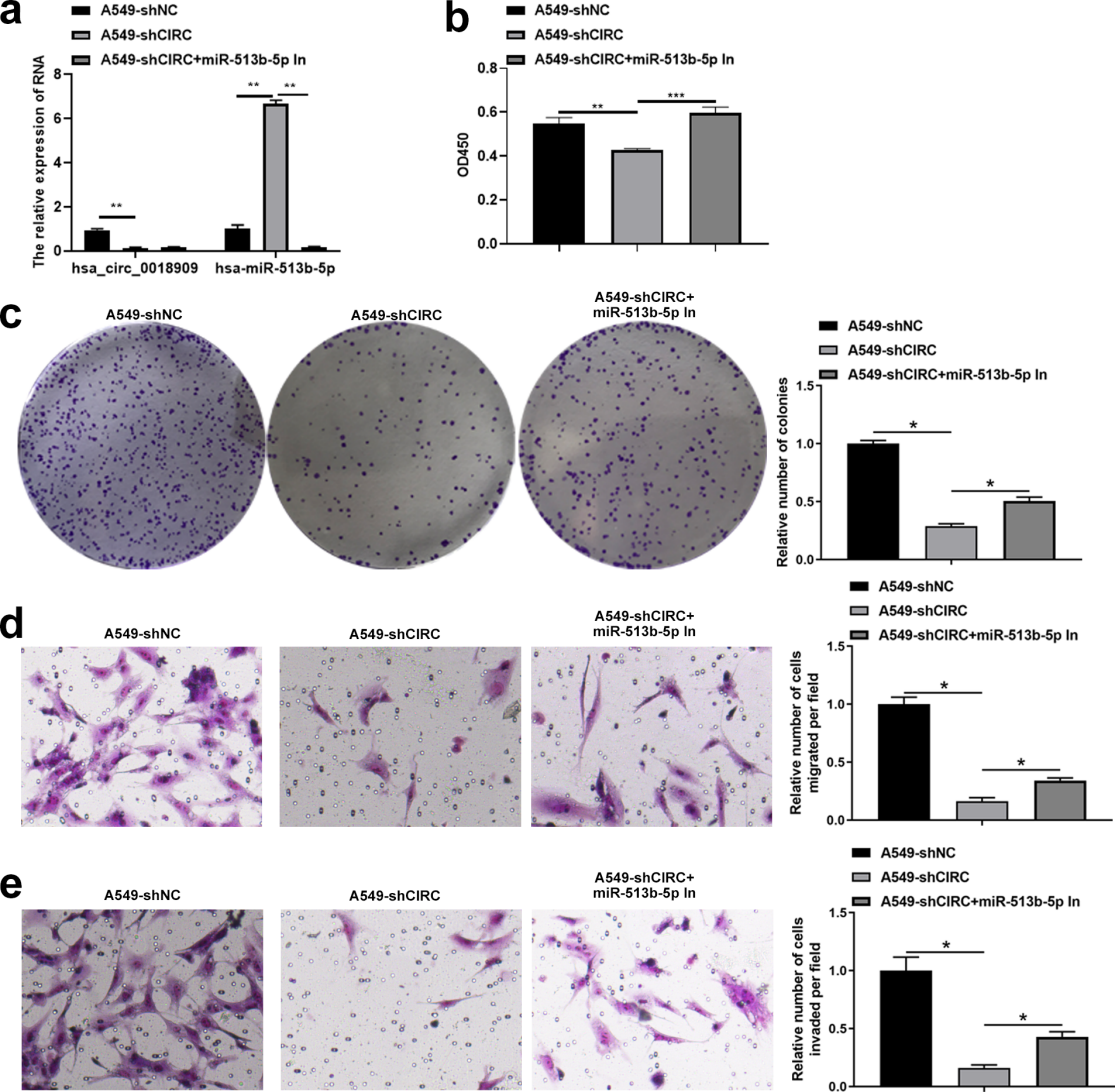
Interference with hsa-miR-513b-5p reversed the effect of shCIRC on the proliferation, migration, and invasion abilities of A549 cells. Inhibitor negative control and hsa-miR-513b-5p inhibitor were transfected into A549-shNC and A549-shCIRC cells for 48 h, respectively. Experimental groups were divided into A549-shNC (A549-shNC cells transfected with inhibitor negative control), A549-shCIRC (A549-shCIRC cells transfected with inhibitor negative control), and A549-shCIRC + miR-513b-5p in (A549-shCIRC cells transfected with hsa-miR-513b-5p inhibitor). **(a)** Detection of tbhsa_circ_0018909 and hsa-miR-513b-5p levels using quantitative reverse transcriptase polymerase chain reaction. **(b)** Cell proliferation was detected using Cell Counting Kit-8 assay. **(c)** Cell proliferation was assessed using a clone formation assay. Cell migration **(d)** and migration **(e)** were determined using a Transwell assay. A549-shCIRC, stable strain with a silenced expression of hsa_circ_0018909; A549-shNC, negative control stable strain with a silenced expression of hsa_circ_0018909. ^*^ *p* < 0.05; ^**^ *p* < 0.01; ^***^ *p* < 0.001.

Next, we investigated whether the downstream target gene hsa-miR-513b-5p, directly regulated by hsa_circ_0018909, could reverse the effects of hsa_circ_0018909 on NSCLC cell proliferation, migration, and invasion. Firstly, hsa-miR-513b-5p mimics were transfected into PC9-LVCIRC cells, and the expression levels of hsa_circ_0018909 and hsa-miR-513b-5p were detected. qRT-PCR results showed that, compared with PC9-LVNC cells, hsa_circ_0018909 expression was significantly upregulated in PC9-LVCIRC cells, while miR-513b-5p expression was significantly downregulated (**Figure 4a**). After overexpression of hsa-miR-513b-5p in PC9-LVCIRC cells, there was no significant change in the expression of hsa_circ_0018909, while the expression of hsa-miR-513b-5p was significantly upregulated (**Figure 4a**). These results indicated that the hsa-miR-513b-5p mimics successfully increased hsa-miR-513b-5p expression and could be used for further cell function experiments. CCK-8 assay results showed that, compared to PC9-LVNC cells, the proliferation of PC9-LVCIRC cells was significantly increased, while hsa-miR-513b-5p mimics attenuated the cell growth rate induced by hsa_circ_0018909 overexpression (**Figure 4b**). Similarly, a consistent trend was observed in the clone formation assay (**Figure 4c**). In addition, overexpression of hsa_circ_0018909 promoted increased migration ability, which was attenuated by hsa-miR-513b-5p mimics (**Figure 4d**). More importantly, a similar effect was observed in the invasion assay (**Figure 4e**). Subsequently, we transfected hsa-miR-513b-5p inhibitor or inhibitor NC into the stable A549-shNC and A549-shCIRC cell lines and divided them into three groups: A549-shNC, A549-shCIRC, and A549-shCIRC + miR-513b-5p inhibitor. The qRT-PCR results showed that, compared to A549-shNC, A549-shCIRC cells exhibited a significant decrease in hsa_circ_0018909 expression and a significant upregulation of hsa-miR-513b-5p (**Figure 5a**). Moreover, the miR-513b-5p inhibitor had no significant effect on the expression of hsa_circ_0018909, while it reduced the expression of hsa-miR-513b-5p in A549-shCIRC cells (**Figure 5a**). These results confirmed that the hsa-miR-513b-5p inhibitor is an effective interfering fragment. The CCK-8 results suggested that shCIRC effectively reduced the cell proliferation ability compared to shNC, while the hsa-miR-513b-5p inhibitor reversed the effect of shCIRC in the A549 cells (**Figure 5b**). The clone formation assay also confirmed such a proliferation ability change (**Figure 5c**). Furthermore, Transwell detected a significant decrease in the migration number of A549-shCIRC cells compared to A549-shNC cells, while the hsa-miR-513b-5p inhibitor increased the migration ability of A549-shCIRC cells (**Figure 5d**). Similarly, the invasive ability of Transwell detection exhibited the same changes as migration (**Figure 5e**). The above results effectively demonstrated that hsa_circ_0018909 controlled the proliferation, migration, and invasion ability of NSCLC cells by directly regulating the expression of hsa-miR-513b-5p.

### 3.7 MDH1 mediates the inhibitory effects of hsa-miR-513b-5p on NSCLC cell proliferation, migration, and invasion

To further clarify the mechanism by which hsa_circ_0018909 contributes to NSCLC progression, we investigated potential downstream targets of hsa-miR-513b-5p. Bioinformatic analysis predicted two putative binding sites for hsa-miR-513b-5p within the 3′ untranslated region (3′UTR) of MDH1 mRNA (**Supplementary Figure S1a**).

qRT-PCR analysis showed that transfection with hsa-miR-513b-5p mimics significantly reduced MDH1 mRNA expression, while inhibition of hsa-miR-513b-5p resulted in upregulation of MDH1 levels (**Supplementary Figure S1b**). Consistent with these results, Western blot assays demonstrated that MDH1 protein expression was downregulated following miR-513b-5p overexpression and upregulated upon its inhibition (**Supplementary Figure S1c**). Moreover, dual-luciferase reporter assays demonstrated that hsa-miR-513b-5p directly interacts with the MDH1 3′UTR, as shown by a significant decrease in luciferase activity in cells transfected with the wild-type construct, but not in those with the mutant construct (**Supplementary Figure S1d**). Collectively, these results confirm that MDH1 is a direct downstream target of hsa-miR-513b-5p in NSCLC cells. In addition, to determine whether MDH1 is functionally involved in the tumor-suppressive effects of hsa-miR-513b-5p, rescue experiments were conducted by co-transfecting A549 cells with si-MDH1 and a hsa-miR-513b-5p inhibitor. qRT-PCR and Western blot analyses confirmed that MDH1 expression was upregulated by miR-513b-5p inhibition and subsequently reduced upon co-transfection with si-MDH1 (**Supplementary Supplementary Figures S2a, b**). The results showed that MDH1 knockdown partially reversed the increase in cell proliferation induced by hsa-miR-513b-5p inhibition (**Supplementary Figures 2c, d**). Similarly, inhibition of miR-513b-5p promoted A549 cell migration and invasion, whereas knockdown of MDH1 attenuated these effects (**Supplementary Figures 2e, f**). These findings suggest that MDH1 may mediate the suppressive role of hsa-miR-513b-5p in NSCLC cell progression.

### 3.8 Hsa_circ_ 0018909 participated in the progression of NSCLC *in vivo*

**Figure 6:**
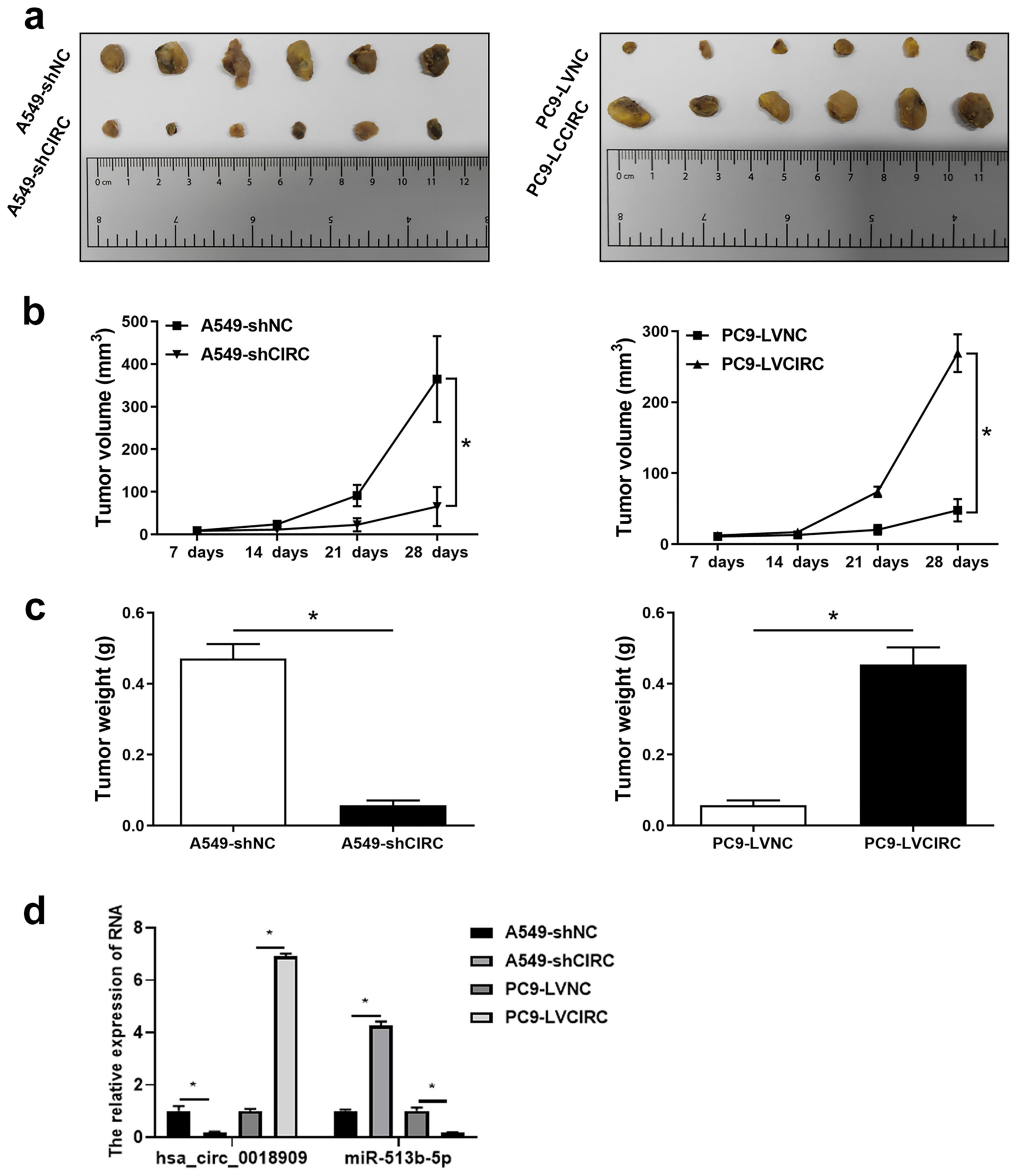
Hsa_circ_ 0018909 participated in the progression of non-small cell lung cancer *in vivo*. A total of 24 nude mice were divided into four groups, with six nude mice per group: A549-shNC, A549-shCIRC, PC9-LVNC, and PC9-LVCIRC. Each mouse received a subcutaneous injection of 3 × 10^6^ cells. **(a)** After 4 weeks, tumor tissues were harvested and photographed to observe their shape and size. **(b)** Tumor volume was measured over the 4-week period. **(c)** Body weight was recorded after 4 weeks. **(d)** Quantitative reverse transcriptase polymerase chain reaction was used to detect hsa_circ_001890 and hsa-miR-513b-5p levels in tumor tissues. A549-shCIRC, stable strain with silenced expression of hsa_circ_0018909; A549-shNC, negative control stable strain with silenced expression of hsa_circ_0018909; PC9-LVCIRC, stable strain with overexpression of hsa_circ_0018909; PC9-LVNC, negative control stable strain with overexpression of hsa_circ_0018909. ^*^ *p* < 0.05.

Finally, we investigated the involvement of hsa_circ_0018909 in the growth of subcutaneous tumors in nude mice. Tumors in the PC9-LVCIRC group were significantly larger in both shape and size compared to the PC9-LVNC group. Conversely, in the interference group (A549-shCIRC), tumor size and volume were significantly reduced compared to the A549-shNC group (**Figure 6a**). Consistent with these observations, tumor volume gradually increased over time across all groups. Over time, tumor volume gradually increased in all groups. However, overexpression of hsa_circ_0018909 significantly promoted tumor growth in the PC9-LVCIRC group compared to the PC9-LVNC group, while knockdown of hsa_circ_0018909 inhibited tumor growth in the A549-shCIRC group compared to the A549-shNC group (**Figure 6b**). Similarly, tumor weight followed the same trend as tumor volume (**Figure 6c**). In addition, expression analysis in tumor tissues showed that overexpression of hsa_circ_001890 led to a decrease in hsa-miR-513b-5p levels, whereas knockdown of hsa_circ_001890 resulted in upregulation of hsa-miR-513b-5p (**Figure 6d**). These results indicate that hsa_circ_001890 promotes subcutaneous tumor growth in NSCLC by regulating hsa-miR-513b-5p expression.

## 4 Discussion

As demonstrated by our findings, hsa_circ_0018909 was significantly upregulated, whereas hsa-miR-513b-5p was notably downregulated in NSCLC tissues and cells, and their expression levels were correlated with poor OS and DFS. Functional assays revealed that overexpression of hsa_circ_0018909 markedly enhanced the proliferation, migration, and invasion of NSCLC cells while silencing hsa_circ_0018909 produced the opposite effects. Moreover, hsa-miR-513b-5p mimics reversed the oncogenic effects induced by hsa_circ_0018909, indicating that hsa_circ_0018909 exerts its tumor-promoting role, at least in part, through suppression of hsa-miR-513b-5p. Mechanistically, we confirmed that hsa_circ_0018909 directly binds to hsa-miR-513b-5p via luciferase reporter assays. Further investigation identified MDH1 as a novel downstream target of hsa-miR-513b-5p. Dual-luciferase assays and rescue experiments demonstrated that MDH1 is directly regulated by hsa-miR-513b-5p, and the knockdown of MDH1 attenuated the enhanced proliferative and invasive capacities induced by miR-513b-5p inhibition. These results suggest that the hsa_circ_0018909/miR-513b-5p/MDH1 axis plays a crucial role in NSCLC progression, providing new insights into the molecular mechanisms underlying NSCLC development. These findings align with previous research showing that circ_0018909 promotes tumor growth and immune evasion, as demonstrated in pancreatic cancer via the miR-545-3p/FASN axis (21). However, this study is the first to reveal the oncogenic function of hsa_circ_0018909 in NSCLC and its regulation of the miR-513b-5p/MDH1 pathway, thereby broadening the understanding of hsa_circ_0018909-mediated mechanisms in NSCLC.

The continuous progress of RNA sequencing technologies, along with bioinformatics tools and databases, has created new opportunities for understanding and functional research of circRNAs (8). The first genome-wide circRNA profiling was reported in 2012 (23), followed by the development of find_circ in 2013, the first publicly available circRNA identification pipeline (24). To date, over a hundred bioinformatics tools related to circRNAs have been developed, supporting circRNA annotation, identification, and ceRNA network analysis (8). The GEO, an international public repository, is widely used by the research community to submit high-throughput microarray and next-generation sequence functional genome datasets (25). In this study, the GSE101586 dataset was used to identify circRNAs upregulated in NSCLC tissues compared to adjacent normal tissues. Subsequently, five upregulated circRNAs were selected for validation using a small clinical sample cohort (10 pairs) and NSCLC cell lines, along with the human normal lung epithelial cell line BEAS-2B. Only hsa_circ_0018909 showed increased expression in NSCLC tissues and cell lines compared to adjacent normal tissues and the BEAS-2B cell line, respectively. Therefore, hsa_circ_0018909 was selected for further functional and mechanistic investigation. The CircBase web server (http://www.circbase.org/) provides information on circRNA sequences and expression, as well as links to UCSC and doRiNA databases (26). In our study, the sequence of hsa_circ_0018909 was downloaded from CircBase, and its chromosomal position was obtained from the UCSC genome browser. The junction site of hsa_circ_0018909 was then confirmed by Sanger sequencing. Additionally, TargetScan (https://www.targetscan.org/vert_80/) predicts the biological targets of miRNAs (27). In this study, miRNAs potentially interacting with hsa_circ_0018909 were predicted using TargetScan and subsequently validated by luciferase reporter assays.

Five circRNAs, including hsa_circ_0000977, hsa_circ_0022383, hsa_circ_0004104, hsa_circ_0018909, and hsa_circ_0001998, were selected for validation in 10 pairs of NSCLC tissues. Notably, Hsa_circ_0000977, also known as circNOL10, has been reported to promote esophageal squamous cell carcinoma progression by sponging miR-874-3p, with its expression upregulated in that cancer type (28). However, circNOL10 expression is downregulated in breast cancer, colorectal cancer, and lung cancer (29–31). Interestingly, circNOL10 is primarily localized in the nucleus, and overexpression of hsa_circ_0000977 inhibits lung cancer development by enhancing SCLM1-mediated transcription regulation of the human peptide family (31). Hsa_circ_0022383, also known as circRNA FADS2, is highly expressed in colorectal cancer and rheumatoid arthritis but downregulated in endometritis (32–34). Hsa_circ_0004104, also referred to as circRNA SPARC, is upregulated in colorectal cancer and promotes cancer cell migration and proliferation through the JAK/STAT pathway (35). Hsa_circ_0001998, also known as circRNA FUT8, is overexpressed in liver cancer and promotes disease progression by sponging miR-548c. In summary, the expression patterns of the same circRNA can vary across different diseases, and a single circRNA may participate in multiple regulatory mechanisms within the same disease.

In our present study, we focused on the function and mechanism of hsa_circ_0018909. Hsa_circ_0018909 is derived from the host gene VDAC2, with the NCBI number NM_001184783. Previous studies have shown that changes in VDAC2 protein levels or posttranslational modifications, such as phosphorylation, occur under various pathological or physiological conditions, including adipogenesis and aging (36). Additionally, VDAC2 plays a role in apoptosis independent of the BCL-2 family, and embryos deficient in VDAC2 exhibit developmental lethality (37, 38). As a partner of the stimulator of interferon response cGAMP interactor 1, VDAC2 participates in nonclassical innate immune signaling regulated by STING, influencing the growth of renal cancer cells (39). However, the exact role and mechanisms of VDAC2 in many cellular functions remain unclear. It is evident that the VDAC2 gene plays an important role in various biological processes. Moreover, few studies have explored the function and mechanism of hsa_circ_0018909 in cancer. Previous studies reported that hsa_circ_0018909 is downregulated in colorectal cancer but upregulated in NSCLC (19, 20). However, its function and underlying mechanisms have not been fully investigated. Only one research publication has indicated that hsa_circ_0018909 promotes pancreatic cell growth and metastasis by regulating miR-545-3p (21). In our present study, we found that hsa_circ_0018909 was overexpressed in NSCLC tissues and cell lines, and its high expression was associated with poor OS and DFS. Overexpression of hsa_circ_0018909 promoted the proliferation, migration, and invasion of NSCLC cells, while its knockdown produced the opposite effects. Similar results were observed in NSCLC cells in nude mouse models. This article is the first to comprehensively investigate the functional role of hsa_circ_0018909 in NSCLC.

To explore the potential mechanism of hsa_circ_0018909, downstream miRNAs were predicted. MiRNAs with two or more binding sites were selected, including hsa-miR-125b-2-3p, hsa-miR-643, hsa-miR-1255b-2-3p, hsa-miR-513b-5p, and hsa-miR-3200-3p. Hsa-miR-125b-2-3p has been reported to be downregulated in colorectal cancer, breast cancer, and liver cancer, and is considered a risk factor for poor prognosis (40–42). Additionally, hsa-miR125b-2-3p functions as a tumor suppressor in breast cancer stem cells (41). However, there are no reports related to hsa-miR125b-2-3p in NSCLC. In this study, we found that overexpression of hsa_circ_0018909 promoted hsa-miR125b-2-3p expression, while silencing hsa_circ_0018909 reduced hsa-miR125b-2-3p expression, suggesting that hsa_circ_0018909 may participate in NSCLC progression by regulating hsa-miR125b-2-3p. Hsa-miR-643 was found to be downregulated in gastric cancer and oral squamous cell carcinoma but upregulated in papillary thyroid carcinoma (43–45). Only one previous study found that hsa-miR-3200-3p was downregulated in glioma (46), and there are currently no reports concerning hsa-miR-1255b-2-3p. In summary, similar to circRNAs, miRNAs may exhibit different expression patterns across various cancer types, and their functions still require further investigation. Our research focused on whether hsa-miR-513b-5p is involved in NSCLC progression through its regulation by hsa_circ_0018909. The results showed that hsa-miR-513b-5p reversed the effects of hsa_circ_0018909 in NSCLC cells. The luciferase assay confirmed the binding sites between hsa_circ_0018909 and hsa-miR-513b-5p. Consistent with previous findings (47), our study also found that hsa-miR-513b-5p expression was reduced in NSCLC tissues. Additionally, we observed that hsa-miR-513b-5p expression was negatively regulated by hsa_circ_0018909, indicating that hsa_circ_0018909 contributes to NSCLC development by directly targeting and regulating hsa-miR-513b-5p. This study is the first to elucidate the function and mechanism of hsa_circ_0018909 in NSCLC, offering a potential new strategy for its treatment. Notably, although miR-545-3p was not among the predicted targets of hsa_circ_0018909 based on our TargetScan analysis in NSCLC, a previous study demonstrated that hsa_circ_0018909 promotes pancreatic cancer progression by regulating miR-545-3p (21). This indicates that circRNA–miRNA interactions may vary depending on the cancer type or biological context. Since miR-545-3p is implicated in key oncogenic processes, including cell proliferation and apoptosis, its potential role in NSCLC warrants further investigation. Future studies using luciferase reporter assays and expression profiling are needed to determine whether hsa_circ_0018909 also exerts regulatory effects through miR-545-3p in the context of lung cancer.

Furthermore, previous studies have reported that hsa-miR-513b-5p functions as a tumor suppressor in various cancers, including liver, pancreatic, ovarian, and gastric cancers (48–51). Our findings are consistent with this tumor-suppressive role and further reveal that miR-513b-5p regulates a novel downstream target, MDH1, in NSCLC—a relationship not previously reported. This suggests that miR-513b-5p may exert context-specific regulatory effects depending on the tumor type, adding a new dimension to its mechanistic landscape in lung cancer.

Importantly, we further explored the downstream regulatory axis of hsa-miR-513b-5p and identified MDH1 as a direct target. Luciferase assays confirmed the binding between hsa-miR-513b-5p and the 3′UTR of MDH1, and functional rescue experiments demonstrated that MDH1 knockdown partially reversed the enhanced proliferation, migration, and invasion caused by hsa-miR-513b-5p inhibition. These findings suggest that hsa_circ_0018909 promotes NSCLC progression, at least in part, through the hsa-miR-513b-5p/MDH1 axis. MDH1 is a cytosolic enzyme involved in the tricarboxylic acid (TCA) cycle, catalyzing the reversible conversion of malate to oxaloacetate (52). It plays a key role in redox homeostasis and energy metabolism (53). Recent studies have demonstrated that dysregulated MDH1 is associated with cancer progression, metabolic reprogramming, and therapy resistance in pancreatic cancer (54), breast cancer (55), and lung adenocarcinoma (56). However, its functional role in NSCLC remains unclear. Our findings provide the first evidence that MDH1 may contribute to NSCLC progression through circRNA/miRNA-mediated regulation, highlighting a novel metabolic node in lung cancer biology.

We found that high expression of hsa_circ_0018909 was associated with smoking history, pathology grade, and lymph node metastasis. Smoking is known to induce widespread genetic and epigenetic changes in lung tissues, promoting tumor initiation and progression (57). It is possible that smoking-related molecular alterations contribute to the upregulation of hsa_circ_0018909 in NSCLC. Furthermore, the positive association between hsa_circ_0018909 expression and higher pathology grade or lymph node metastasis suggests that hsa_circ_0018909 may be involved in regulating tumor aggressiveness and metastatic potential. These findings imply that hsa_circ_0018909 might not only serve as a diagnostic biomarker but also reflect the malignant progression of NSCLC. Further mechanistic studies are warranted to explore the detailed pathways underlying these clinical correlations.

Despite these findings, several limitations of this study should be acknowledged. First, the downstream targets of hsa-miR-513b-5p and the biological functions of hsa-miR-125b-2-3p were not comprehensively investigated. Further studies are required to elucidate the regulatory networks mediated by these miRNAs through both *in vivo* and *in vitro* experiments. Second, detailed treatment information, including surgical procedures, chemotherapy, and radiotherapy, was not systematically collected for all patients, which may have impacted the survival analyses. These limitations emphasize the need for future research involving cohorts with complete clinical and therapeutic data to refine the prognostic evaluation of hsa_circ_0018909. In addition, future work should incorporate multicenter cohorts with larger sample sizes to validate our findings and further explore the clinical significance of the hsa_circ_0018909/miR-513b-5p/MDH1 axis. Collectively, our study advances the theoretical understanding of circRNA-miRNA-mRNA regulatory networks in NSCLC and provides practical implications for biomarker discovery and targeted therapeutic strategies.

## 5 Conclusion

Altogether, hsa_circ_0018909 was highly expression, whereas hsa-miR-513b-5p showed low expression in NSCLC tissues. Their expression levels were associated with poor OS and DFS. Hsa_circ_0018909 directly binds with hsa-miR-513b-5p to participate in the progression of NSCLC. Overexpression of hsa_circ_0018909 increased cell proliferation, migration, and invasion abilities, while hsa-miR-513b-5p mimics reversed the effects of hsa_circ_0018909 on cell function. Knockdown of hsa_circ_0018909 produced opposite results. Our study is the first to investigate the mechanism of hsa_circ_0018909 in NSCLC, providing new insights for NSCLC treatment.

## Data Availability

The original contributions presented in the study are included in the article/**Supplementary Material**. Further inquiries can be directed to the corresponding author.

## Supplementary material

Supplementary Figure 1 Validation of MDH1 as a direct target of hsa-miR-513b-5p. **(a)** Predicted binding sites between hsa-miR-513b-5p and two regions within the (3’UTR of MDH1 based on bioinformatic analysis. **(b)** qRT-PCR analysis of MDH1 mRNA expression levels in A549 cells after transfection with hsa-miR-513b-5p mimics or inhibitors. **(c)** Western blot analysis showing consistent changes in MDH1 protein expression following manipulation of hsa-miR-513b-5p levels. **(d)** Dual-luciferase reporter assay assessing the interaction between hsa-miR-513b-5p and the wild-type or mutant MDH1 3’UTR constructs. *p < 0.05, **p < 0.01.

Supplementary Figure 2 MDH1 mediates the tumor-suppressive effects of hsa-miR-513b-5p in NSCLC cells. **(a)** qRT-PCR analysis of MDH1 s in A549 cells transfected with hsa-miR-513b-5p inhibitor alone or co-transfected with si-MDH1. **(b)** Western blot analysis of MDH1 protein expression following transfection with hsa-miR-513b-5p inhibitor or combined si-MDH1 and hsa-miR-513b-5p inhibitor. **(c)** Cell proliferation was assessed by CCK-8 in the indicated treatment groups. **(d)** A colony formation assay was conducted to evaluate the proliferative capacity of A549 cells under different transfection conditions. **(e, f)** Transwell assay was performed to assess the migratory and invasive ability of A549 cells. *p < 0.05, **p < 0.01, ***p < 0.001.
