# Supplementary figures and images for: Hsa_circ_0018909 promotes non-small cell lung cancer by directly regulating hsa-miR-513b-5p
Source: Front Oncol. 2025 Jun 23;15:1542742. doi: 10.3389/fonc.2025.1542742 (PMC12230065; doi:10.3389/fonc.2025.1542742)

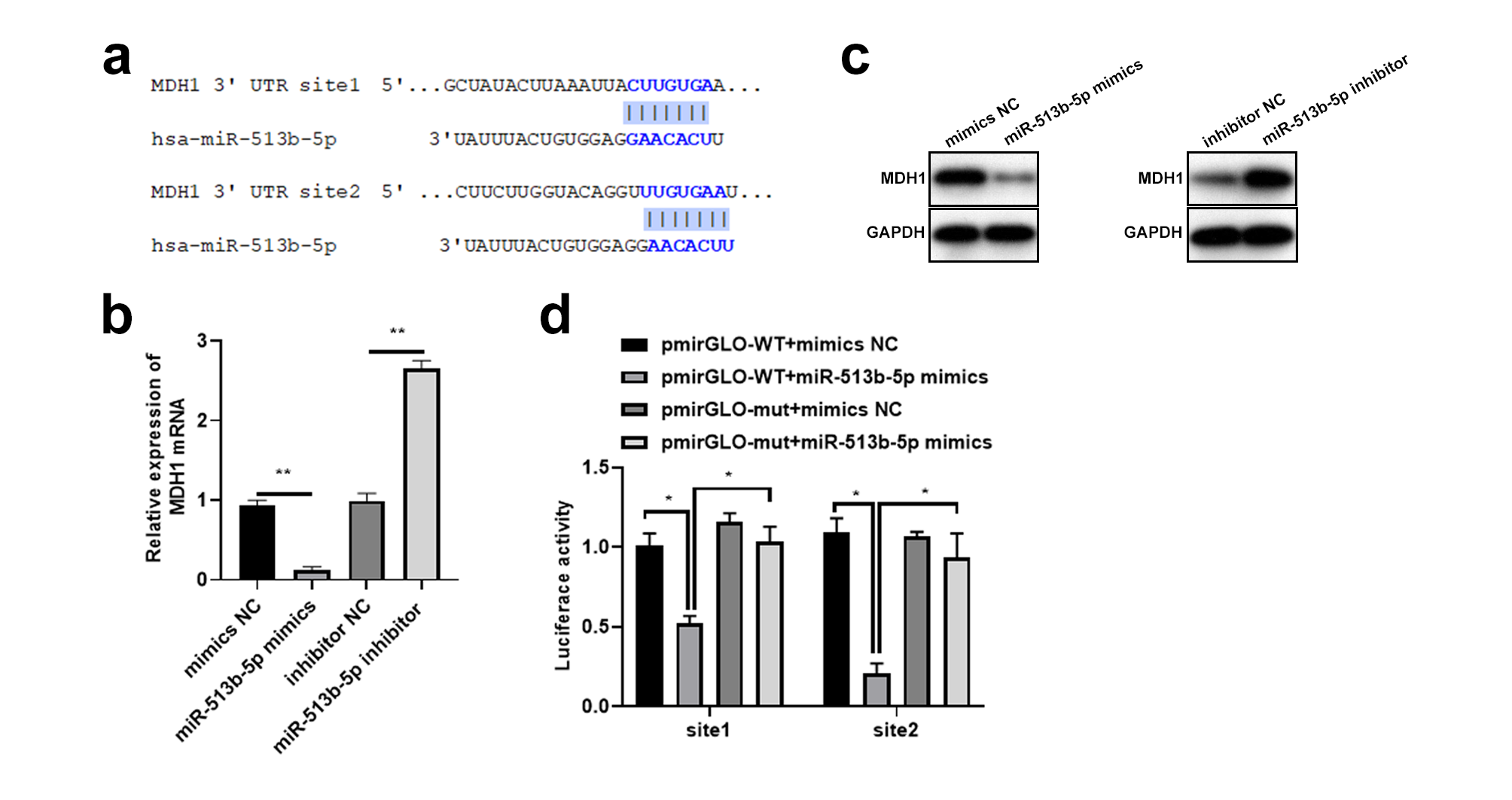

Supplement: Supplementary Figure 1 — Validation of MDH1 as a direct target of hsa-miR-513b-5p. (a) Predicted binding sites between hsa-miR-513b-5p and two regions within the (3’UTR of MDH1 based on bioinformatic analysis. (b) qRT-PCR analysis of MDH1 mRNA expression levels in A549 cells after transfection with hsa-miR-513b-5p mimics or inhibitors. (c) Western blot analysis showing consistent changes in MDH1 protein expression following manipulation of hsa-miR-513b-5p levels. (d) Dual-luciferase reporter assay assessing the interaction between hsa-miR-513b-5p and the wild-type or mutant MDH1 3’UTR constructs. *p < 0.05, **p < 0.01. [file Image1.tif]

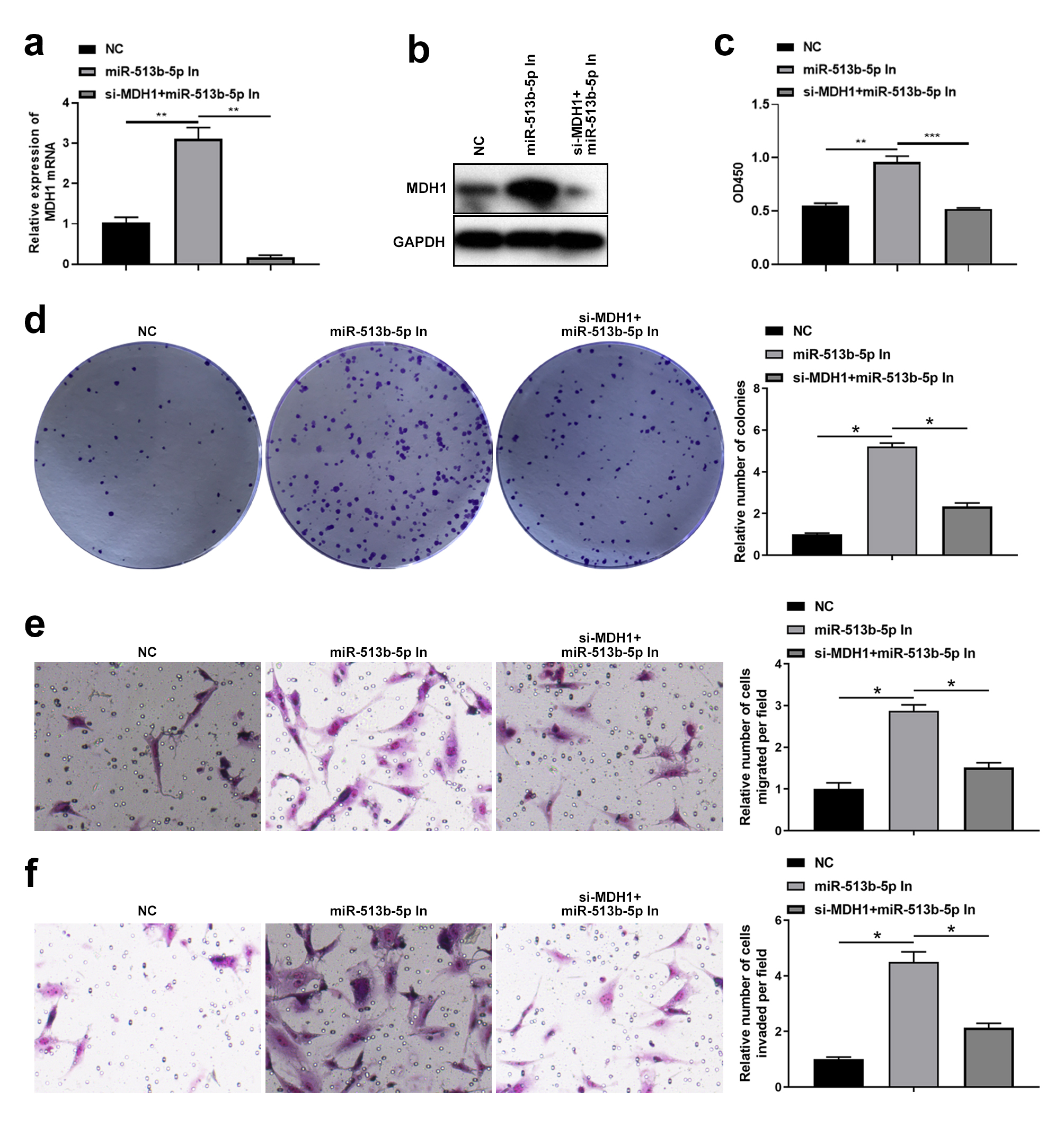

Supplement: Supplementary Figure 2 — MDH1 mediates the tumor-suppressive effects of hsa-miR-513b-5p in NSCLC cells. (a) qRT-PCR analysis of MDH1 s in A549 cells transfected with hsa-miR-513b-5p inhibitor alone or co-transfected with si-MDH1. (b) Western blot analysis of MDH1 protein expression following transfection with hsa-miR-513b-5p inhibitor or combined si-MDH1 and hsa-miR-513b-5p inhibitor. (c) Cell proliferation was assessed by CCK-8 in the indicated treatment groups. (d) A colony formation assay was conducted to evaluate the proliferative capacity of A549 cells under different transfection conditions. (e, f) Transwell assay was performed to assess the migratory and invasive ability of A549 cells. *p < 0.05, **p < 0.01, ***p < 0.001. [file Image2.jpg]
